# Feasibility and Acceptability of the Technology-Based Fall Risk Assessments for Older Adults

**DOI:** 10.1093/geroni/igab046.3603

**Published:** 2021-12-17

**Authors:** Ladda Thiamwong, Oscar Garcia, Renoa Choudhury, Joon-Hyuk Park, Jeffrey Stout, Rui Xie

**Affiliations:** 1 College of Nursing, University of Central Florida, Orlando, Florida, United States; 2 University of Central Florida, Orlando, Florida, United States

## Abstract

Promising technologies, which are simple, portable, quick, non-invasive, and inexpensive, may open new horizons on fall risk assessments and provide important information for older adults. We used a mixed-methods approach to examine the feasibility and acceptability of technology-based fall risk assessments, including the BTrackS Balance System, Bioelectrical Impedance Analysis, and activity monitoring devices among older adults. Data were collected via a Qualtrics survey and interviews. The acceptability was measured by the Senior Technology Acceptance (STA) and semi-structured interviews with 15 participants. The STA consists of four domains with 14 items, and the semi-structured interview includes three main questions related to experiences about balance performance tests, body composition, and activity monitoring. One hundred twenty-four community-dwelling older adults completed the online survey, and 15 older adults participated in the interviews. The majority of participants were female, and 72% had no history of falls. Race and ethnicity were 17% Hispanic, 7% African Americans, and 3% Asian Americans. About 7% had COVID-19 positive, 31% reported fear of COVID, and 14.5% were afraid of losing their life to COVID. The word-of-mouth strategy and key person approach were used and had an incredible impact on the recruitment process. None of the participants had ever had their fall risk and fear of falling assessed before agreeing to participate in this study. The technology-based fall risk assessments were feasible and acceptable. About 78% of participants liked the idea of using technology to assess falls risk, and 79% agreed that using technology would enhance their effectiveness in daily activities.

